# Development of the Observation Schedule for Children with Autism–Anxiety, Behaviour and Parenting (OSCA–ABP): A New Measure of Child and Parenting Behavior for Use with Young Autistic Children

**DOI:** 10.1007/s10803-020-04506-3

**Published:** 2020-04-30

**Authors:** Melanie Palmer, Juan Paris Perez, Joanne Tarver, Thomas Cawthorne, Margot Frayne, Sophie Webb, Elena Baker, Isabel Yorke, Dale Hay, Vicky Slonims, Andrew Pickles, Emily Simonoff, Stephen Scott, Tony Charman

**Affiliations:** 1 Department of Child and Adolescent Psychiatry, King’s College London, Institute of Psychiatry, Psychology & Neuroscience, London, UK; 2 Department of Psychology, School of Life and Health Sciences, Aston University, Birmingham, UK; 3 Department of Psychology, King’s College London, Institute of Psychiatry, Psychology & Neuroscience, London, UK; 4 School of Psychology, Cardiff University, Cardiff, UK; 5 Newcomen Neurodevelopmental Centre, Evelina Children’s Hospital, Guy’s and St Thomas NHS Foundation Trust, London, UK; 6 Department of Biostatistics and Health Informatics, King’s College London, Institute of Psychiatry, Psychology & Neuroscience, London, UK; 7 Service for Complex Autism & Associated Neurodevelopmental Disorders, South London and Maudsley NHS Foundation Trust, London, UK

**Keywords:** Autism, Child emotional and behavioral problems, Parenting, Measurement, Observation

## Abstract

Co-occurring emotional and behavioral problems (EBPs) frequently exist in young autistic children. There is evidence based on parental report that parenting interventions reduce child EBPs. More objective measures of child EBPs should supplement parent reported outcomes in trials. We describe the development of a new measure of child and parenting behavior, the Observation Schedule for Children with Autism–Anxiety, Behaviour and Parenting (OSCA–ABP). Participants were 83 parents/carers and their 4–8-year-old autistic children. The measure demonstrated good variance and potential sensitivity to change. Child and parenting behavior were reliably coded among verbal and minimally verbal children. Associations between reports from other informants and observed behavior showed the measure had sufficient convergent validity. The measure has promise to contribute to research and clinical practice in autism mental health beyond objective measurement in trials.

**Electronic supplementary material:**

The online version of this article (10.1007/s10803-020-04506-3) contains supplementary material, which is available to authorized users.

Autism^1^ is characterized by impaired social and communication skills, the presence of restrictive and repetitive interests and behaviors and sensory anomalies (American Psychiatric Association 2013), occurring in approximately 1% of the population (Baio et al. 2018; Baird et al. 2006). Emotional and behavioral problems (EBPs) are also common in autistic individuals (Lai et al. 2019); with up to as many as 90% of children and adolescents meeting diagnostic criteria for an anxiety disorder, attention deficit hyperactivity disorder (ADHD), oppositional defiant disorder (ODD) or conduct disorder (Salazar et al. 2015; Simonoff et al. 2008; White et al. 2009). For autistic individuals, EBPs tend to persist over time (Simonoff et al. 2013) and impact on their quality of life (Mason et al. 2018). Parents often report that they would like support with these co-occurring difficulties, which are associated with lower parental wellbeing and more parental stress (Yorke et al. 2018). One challenge in research and clinical settings is obtaining accurate information about the severity of child EBPs, triggers and parental management strategies. Often parent and teacher reports are relied on, which have certain biases, and more objective measures are needed.

One of the most established psychosocial approaches for improving EBPs in non-autistic populations are behavioral parenting interventions (National Institute for Health and Clinical Excellence 2013). A large literature has demonstrated that increases in child-centered parenting (e.g. praise, positive comments, child-led play) are associated with fewer child EBPs, whereas controlling and critical parenting behaviors are associated with worsening child EBPs (e.g. Patterson 1982). Behavioral parenting interventions aim to improve child behavior through modification of these parenting behaviors and improvement of the parent–child relationship (Barlow et al. 2016). Although behavioral parenting interventions derive from operant conditioning and social learning theories, rather than attachment-based approaches, such interventions also promote parents’ sensitive responding to their children (O'Connor et al. 2013).

Such interventions have been adapted for parents of autistic children. Adaptations have included accounting for differing mechanisms, such as anxiety and sensory processing, that might underlie EBPs in autism (Bearss et al. 2015b; Green et al. 2012; Ozsivadjian et al. 2012; Tseng et al. 2011), as well as different responses to strategies, such as time-out, that are typically included in behavioral parenting interventions (Dababnah and Parish 2016). In comparison to non-autistic samples, fewer investigations have examined the relationship between parenting behaviors and EBPs in autism, although there is evidence that parental criticism is also linked with behavioral problems in this population (Romero-Gonzalez et al. 2018). Other research has identified common parenting behaviors that may be specific to parenting a child with autism, such as accommodating for the child, modifying the environment, stimulating development, providing structure and familiarity and being vigilant (Joosten and Safe 2014; Lambrechts et al. 2011; O'Nions et al. 2018).

Randomized controlled trials (RCTs) examining the efficacy of parenting interventions in autistic populations have demonstrated promising findings (Postorino et al. 2017; Tarver et al. 2019). When using parental reports of EBPs as outcomes, behavioral parenting interventions have been found to reduce disruptive behavior and hyperactivity. While the parent’s view of child EBPs is a clinically relevant and important outcome to be considered following intervention, parent-reported outcomes carry several limitations. One source of bias in trials is knowledge of intervention allocation (Aspland and Gardner 2003), which can impact on effect sizes, usually inflating benefits. The importance of supplementing parent-reported outcomes with blinded measures has been highlighted in the ADHD literature, where a meta-analysis of psychosocial interventions found that effect sizes for behavioral interventions were near-zero when considering outcomes from informants ‘probably-blind’ to intervention status; in contrast, when reports by those closest to the therapeutic setting were used (i.e. parent or teacher) a small-to-medium effect (*SMD* = 0.40) was found (Sonuga-Barke et al. 2013). Other sources of bias in parental reports include broader rater effects, such as mood or stress, or misinterpretation of questionnaire items (Handen et al. 2013). These biases are likely to present across different intervention arms and possibly be influenced by intervention, reducing measurement precision.

Direct observational measures have some advantages over parent-reported outcomes. Items can be defined by researchers and measured to an agreed level of reliability, reducing and quantifying potential inter-reporter variability (Aspland and Gardner 2003). In trials of parenting interventions, child and parenting behaviors of interest can therefore be consistently coded by assessors who are blind to intervention status of the participant. Several observations of parent–child interaction based on social learning and behavioral theories have been developed for use in non-autistic samples. They include the Dyadic Parent–Child Interaction Coding System (Robinson and Eyberg 1981), the Family Observation Schedule (Sanders et al. 1996), the Disruptive Behavior Diagnostic Observation Schedule (Wakschlag, Hill et al. 2008a, b), the Anxiety Dimensional Observation Schedule (Mian et al. 2015) and the Preschool Observational Scale of Anxiety (Glennon and Weisz 1978). These measures have demonstrated good inter-rater and test–retest reliability. Literature examining convergent validity of observational measures has reported small but significant associations between parent-reported and observed behavior in children and parents (e.g. Hawes and Dadds 2006; Wakschlag, Briggs-Gowan et al. 2008a, b).

Direct observational measures of EBPs have scarcely been used in parenting intervention trials in autism to date (Tarver et al. 2019). Where observational measures have been used, floor effects have been reported. Using the Structured Observational Analog Procedure—a parent–child interaction measure consisting of a series of four 10-min conditions (free play, social attention, demand, tangible restriction)—autistic children who were screened in on elevated levels of EBPs displayed unexpectedly high levels of compliance during the clinic-based assessment at baseline, limiting scope for change on this measure (Bearss et al. 2015a; Handen et al. 2013). High rates of compliance at baseline have also been reported on the DPICS, which consists of child- and parent-led play conditions and a clean up (Scudder et al. 2019). Similarly, Tellegan and Sanders (2014) noted that few observed aversive parenting behaviors were seen during 30-min observations of parent–child interactions conducted in the home. One reason for these floor effects may be due to situational factors that influence behavioral presentations, which may in turn reduce effect sizes. This could be especially true for autism where idiosyncratic triggers for EBPs are commonplace (Bearss et al. 2015b; Ozsivadjian et al. 2012). It is also possible that existing observational measures of parent–child interaction are tapping everyday situations that non-autistic children may find difficult (e.g. removal of social attention), but may elicit different reactions from autistic children. Furthermore, Williams et al. (2020) found that some parents found the child-led play component of the DPICS challenging due to difficulties in getting their autistic child to engage. Measures may therefore require modification in order to be reliable and valid assessments of child and parenting behavior in autism.

The current study aimed to develop a measure to observe child and parenting behaviors suitable for use with young autistic children. The Observation Schedule for Children with Autism–Anxiety, Behaviour and Parenting (OSCA–ABP) was developed in the context of a trial of novel group parenting interventions (Palmer et al. 2019) and designed to include everyday situations that autistic children and their parents may face and find difficult. In this article, we report data on the intital reliability and validity of the measure. We examined variability in the scores on the measure, inter-rater reliability and convergent validity by exploring the relationships between observed behaviors and questionnaires completed by parents and teachers.

## Method

### Participants

The sample consisted of 83 parents/carers (mothers: *n* = 76, 91.6%; fathers: *n* = 5, 6.0% grandmothers: *n* = 2, 2.4%) and their 4- to 8-year-old autistic child, recruited through local autism diagnostic teams, education professionals, support groups and consented databases in four boroughs in South London (Bromley, Croydon, Lambeth and Lewisham). Children were on average 6.7 years old (*SD* = 1.21) and most of the sample were male (*n* = 71, 85.5%). Thirty-nine (47.0%) of the children were minimally verbal (defined as non-verbal or having single words), with the remainder (*n* = 44, 53.0%) defined as verbal (flexible phrase or fluent speech). Eleven children (13.1%) had also been diagnosed with ADHD, two with ODD (2.4%) and one with an anxiety disorder (1.1%), as reported by parents. Seven children (8.3%) were prescribed a psychostimulant and three (3.6%) were prescribed an antipsychotic. Further information about the children and their parents is displayed in Table 1.

**Table 1:** Demographic information by child verbal ability group

Demographic characteristics	Verbal (*N* = 44)		Minimally verbal (*N* = 39)	
	*n *or *M*	% or *SD*	*N *or *M*	% or *SD*
Child age (mean years)	7.21	0.83	6.13	1.32
Child gender				
Male	36	81.8%	35	89.7%
Female	8	18.2%	4	10.3%
Child ethnicity^a^				
White	30	69.8%	14	36.8%
Black/Black British	3	7.0%	11	28.9%
Asian/Asian British	3	7.0%	5	13.2%
Mixed/multiple ethnicities	7	16.3%	8	21.1%
Child education type^b^				
Mainstream school	33	76.7%	4	10.3%
Specialist unit in mainstream school	6	14.0%	7	18.0%
Specialist school	4	9.3%	28	71.8%
Parental education level^c^				
No formal qualifications	3	7.0%	7	18.0%
General certificate of secondary education or equivalent	7	16.3%	2	5.1%
General certificate of education advanced level (A levels) or equivalent	7	16.3%	3	7.7%
Vocational qualifications (NVQ, City and guilds or equivalent)	9	20.9%	4	10.3%
Undergraduate tertiary degree	6	14.0%	10	25.6%
Postgraduate tertiary degree	11	25.6%	13	33.3%
Parental employment status^b^				
Not in paid employment	21	48.8%	19	48.7%
In part-time paid employment	14	32.6%	11	28.2%
In full-time paid employment	8	18.6%	9	23.1%
Annual household income^d^				
Less than £20,000	13	39.4%	10	32.3%
£20,000–£39,999	7	21.2%	7	22.6%
£40,000–£59,999	5	15.2%	3	9.7%
£60,000–£79,999	2	6.1%	8	25.8%
Greater than £80,000	6	18.2%	3	9.7%

Valid % reported

^a^*n *= 43 for verbal children, *n *= 38 for minimally verbal children. White = English/Welsh/Scottish/Northern Irish/Irish/British/Other White ethnicity, Black/Black British = African/Caribbean/Other Black ethnicity, Asian/Asian British = Indian/Pakistani/Bangladeshi/Chinese/Other Asian ethnicity, Mixed/Multiple ethnicities = White and Black Caribbean/White and Black African/White and Asian/Other Mixed ethnicity

^b^*n *= 43 for verbal children, *n *= 39 for minimally verbal children. Mainstream school = education setting for children who access the mainstream curriculum, Specialist unit in mainstream school = resource base within a mainstream school for children who access the mainstream curriculum whilst having specialist intervention support, Specialist school = education setting for children with special educational needs or disabilities. Employment status is described for parent/carer involved in completing the observation

^c^*n *= 43 for verbal children, *n *= 39 for minimally verbal children

^d^*n *= 33 for verbal children, *n *= 31 for minimally verbal children

### Procedure

The measure was developed in the context of a trial testing a group-based parenting intervention targeting EBPs as part of the Improving Autism Mental Health (IAMHealth) research programme (https://iamhealthkcl.net). The Autism Spectrum Treatment and Resilience (ASTAR) study consisted of two phases: a non-randomized feasibility study followed by a pilot RCT. Twenty-one parent–child dyads participated in the feasibility study and 62 participated in the pilot RCT. Only data collected during the baseline assessments from both phases was used in the current study. Information on the outcome of the trial, including the measure’s sensitivity to change, will be reported in a separate manuscript.

Children with an existing clinical diagnosis of an autism spectrum disorder were referred into the trial via local autism diagnostic teams, education professionals and support groups at participating services. Potential participants could also self-refer. In order to be eligible to take part, the children were required to have a clinical diagnosis of an autism spectrum disorder (including those classified by ICD-10 criteria: childhood autism, Asperger’s syndrome, pervasive developmental disorder or atypical autism) and be between 4 years 0 months and 8 years 11 months. Given the high prevalence of co-occurring EBPs in autistic children, there was no specific cut-off for inclusion in the trial based on the levels of EBPs children displayed. Families were excluded if: they did not have sufficient spoken English to be able to take part in a group intervention; the child or parent had a severe hearing or visual impairment; the child had seizures more than once a week; there were active safeguarding concerns; or the parent had a current severe psychiatric disorder (see https://www.isrctn.com/ISRCTN91411078 for the trial record).

Written informed consent was obtained from all participating parents/carers and child assent was obtained wherever appropriate. All observations were video-recorded and most (*n* = 79, 95.2%) were completed at a research center in our child-friendly family room. Three observations were conducted at the child’s school and one at a local clinic setting familiar to the child. In these cases, parents did not agree to completing the observation in an unfamiliar environment, due to the child’s anticipated challenging behavior or anxiety. After the assessment, parents were asked to comment briefly on how typical their child’s and their own behavior was during the observation and a brief description of their feedback is in the supplementary materials. In addition, direct assessment of the child’s autism traits were completed. Parent report questionnaire measures of the child’s autism characteristics and functioning, their EBPs, and their experiences of parenting a child with autism were also obtained. With parental consent, the child’s teacher was also asked to complete questionnaires about the child’s EBPs at school.

### Development of the Observation Schedule for Children with Autism Spectrum Disorders–Anxiety, Behaviour and Parenting (OSCA-ABP)

#### Patient and Public Involvement (PPI)

Panels of autistic adults and parents of autistic children have been involved in all phases of the study and assisted with the development of the task schedule (e.g. advising on how to make specific tasks effective at eliciting EBPs). The tasks aimed to simulate everyday challenges that autistic children may face and find difficult, drawing on and modifying existing observational measures of parent–child interaction. Guidance and advice about which behaviors to code was given, particularly in relation to behavioral manifestations of anxiety, as well as assistance with the interpretation of the results. Prior to deciding the final task schedule, a range of tasks were piloted with 12 parents and their 4 to 8-year-old autistic child and 11 parents with non-autistic children of the same age. Feedback was sought on the tasks and materials, the clarity of the instructions and visual prompts, the order of the activities and suggestions for improvement. Tasks that did not appear effective in eliciting challenging behaviors or anxiety were removed (e.g. suspense games including a jack-in-the-box, Buckaroo!^©^ and Jenga^©^ along with an unexpected alarming noise). Removal of these tasks made the measure shorter and more feasible to administer.

Generally, parents thought the length of the measure was acceptable, that the instructions were clear and that the games and activities were suitable (i.e. good variety, gender neutral, covering a range of developmental skills). Some parents of autistic children commented these were activities that would likely lead to EBPs and that behaviors displayed during the observation were generally typical of their child, providing some initial evidence for ecological validity. Parental suggestions for modification of activities to observe challenging behavior were considered in relation to the suitability of administration in a controlled research environment and appropriate modifications were incorporated. For example, removal of a favorite toy or desired object such as a tablet was frequently suggested by parents, but this activity was not included in the observation due to difficulty in finding equally salient objects and replicating this from one child to the next.

#### Final Task Schedule

The measure consisted of two researcher-led and six parent-led tasks. The tasks aimed to elicit observable child behaviors that challenge (BTC) by tapping into uncertainty and novelty, transition, turn taking, sensory processing, compliance, frustration and reward delay. It is designed to be conducted in an environment unfamiliar to the family, to further elicit child BTC. A buzzer is used to signal to the parent to transition their child on to the next activity adding further uncertainty as task duration is managed by the measure administrator. The specific materials used in each task are differentiated by child verbal ability group based on the Autism Diagnostic Observation Schedule—2nd edition (ADOS–2, Lord et al. 2012) assessment modules, which takes into account expressive language and age but were analogous in function (e.g. the shared game either involved playing together on the Simon^©^, a 2-arm Bop It^©^ or a 4-arm Bop It^©^). Table 2 describes the specific tasks and materials used. The duration of the observation aimed to be around 18 to 22 min. Further information about the administration of tasks can be obtained by contacting the corresponding author.

**Table 2:** Task schedule for the OSCA–ABP

	Tasks			Led by	Length (min) total duration ranges from ~ 18 to 22 min
	Minimally verbal—none to some words (ADOS–2 Module 1)	Verbal—phrase speech (ADOS–2 Module 2)	Verbal—fluent speech (ADOS–2 Module 3)		
1.	Mystery box and blindfold			Researcher	Maximum 2 min
2.	Shared task (stacking cups)	Shared task (drawing a square on the Etch A Sketch^©^)	Shared task (drawing a house on the Etch A Sketch^©^)	Parent	2–3 min
3.	Shared game (Simon^©^)	Shared game (2-arm Bop It^©^)	Shared game (4-arm Bop It^©^)	Parent	2–3 min
4.	Separation from parent and free play with Duplo^©^/Lego^©^ and puzzles, dependent on developmental level			Parent → Researcher	Maximum 3 min
5.	Reunification with parent and tidy away the toys			Parent	Maximum 2 min
6.	Homework sheet, adjusted to developmental level with increasing difficulty			Parent	3–5 min
7.	Taking shoes and socks off and walking heel to toe along a line on the floor			Parent	Maximum 2 min
8.	Snack jar with glued lid, with a different unglued jar provided after short delay			Researcher	Maximum 2 min

#### Coding Scheme Development

As the measure was developed within the context of a pilot RCT testing a parenting intervention aiming to reduce EBPs, behaviors that were anticipated to change if such an intervention was effective were the primary focus of the coding scheme. Using a social learning theory lens, we wanted to capture a range of child behaviors that challenge others, such as non-compliant or aggressive behavior, as well as behavioral manifestations of anxiety (e.g. avoidance, Bearss et al. 2015b; Ozsivadjian et al. 2012) and potentially maladaptive coping strategies that may challenge others in certain contexts (e.g. reassurance seeking). Furthermore, we wanted to capture compliance with parental and researcher commands. We considered coding compliance in relation to the type of command (e.g. clear, unclear; see supplementary material Table 3 for definitions) given by the interaction partner but instead used a basic frequency count to assist with obtaining sufficient inter-rater reliability. In addition, a variety of parenting behaviors that have been associated with the absence or presence of EBPs in non-autistic children were included (termed facilitative and non-facilitative parenting respectively).

Using videos of the measure from the piloting phase, definitions of relevant child and parenting behavior items were developed, adapted and clarified through discussion by MP, JT and JPP. The final coding scheme consisted of several child and parenting items that were aggregated into a priori defined domain scores. Because externalizing behavior is often reported as being related to anxiety in autism (Bearss et al. 2015b), these behaviors were grouped together. Clear parental commands were included in facilitative parenting behavior as positively stated clear commands are thought to be helpful for children with disabilities (Green et al. 2014; Marfo 1990). This domain designated desirable parenting and the inclusion of clear commands here is in contrast with some other coding schemes (e.g. Scott et al. 2010) where all commands are included under non-facilitative parenting. The supplementary materials contains the definitions of the items that make up the domain scores described below, as well as information on items that do not form part of the child and parent domain scores but measure other aspects of child and parenting behaviors and global impressions across the observation.

We were interested in establishing whether we could reliability code frequency counts of specific child and parent behaviors separately and did not link child and parent behavior. To establish whether the measure had sufficient inter-rater reliably, we wanted to demonstrate whether general patterns of behavior during the observation were consistent across different coders (i.e. two different coders both rated the same child as displaying a high occurrence of behaviors that challenge). The observations were coded from the video-recordings by at least two raters (JPP, MF, SW, EB) over two viewings (one to code child behavior and the other parenting behavior).

For the children, the frequencies of a range of behaviors (destructive behavior, aggression towards themselves and others, frustrated vocalisations, non-compliance, avoidance and reassurance seeking) observed during the OSCA–ABP were coded and summed to produce the total child BTC score. As the duration of the measure varied, the rate of child BTC per minute is calculated by dividing the total BTC count by the duration. In addition, the frequency of observed child compliance is coded and the rate of child compliance per minute is also calculated.

For the parents, frequencies of observed facilitative parenting (positive comments, clear commands, praise and supportive physical guidance) and non-facilitative parenting (negative comments, unclear commands, no opportunity to comply and physical handling) were coded and summed to produce total facilitative parenting behavior and non-facilitative parenting behavior scores. As with the child domains, rates of facilitative and non-facilitative parenting behaviors were calculated. To account for absolute levels of parenting behaviors, a proportion of facilitative parenting behavior was also calculated to determine relative levels. This was done by dividing the frequency of facilitative parenting by all parenting behaviors (facilitative + non-facilitative).

### Sample Characterisation Measures

Demographic information about the family was obtained using a questionnaire developed for the study.

To characterise the sample, measures of autism severity and adaptive behavior were obtained. Autism severity was measured using the ADOS–2 (Lord et al. 2012), the gold standard observation for assessing autism characteristics administered by four of the authors who were researchers trained to research reliability. Parent report of autism severity was measured using the Social Communication Questionnaire-Lifetime version (SCQ), a 40 item questionnaire measuring the presence of autistic symptoms using a *yes–no* format (Rutter et al. 2003). Scores greater than or equal to 15 is the cut-off point for autism spectrum disorder. There were no criteria for inclusion in the study based on the ADOS–2 or SCQ scores.

To measure adaptive skills and functioning the Adaptive Behavior Assessment System–3rd edition (ABAS–3, Harrison and Oakland 2015) was completed by parents. Both the 0–5-year-old (241 items covering 10 areas) and the 5–21-year-old (211 items covering nine areas) versions were used according to the child’s chronological age and functioning level. A standardized General Adaptive Composite (GAC) score was calculated. Scores for the sample on the ADOS–2, SCQ and the ABAS–3 are presented in the supplementary materials.

### Parent-Reported Child Emotional and Behavioral Problems

The Aberrant Behavior Checklist (ABC) Irritability (15 items) and Hyperactivity (16 items) subscales were used to measure parent-rated child EBPs (Aman et al. 1985). Items were rated on a 4-point scale ranging from ‘*not at all a problem*’ to ‘*the problem is severe in degree*’ with higher scores signifying more EBPs.

Child non-compliance was measured using the Home Situations Questionnaire-Autism Spectrum Disorders (HSQ-ASD, Chowdhury et al. 2015), an autism-specific measure of non-compliance in everyday situations. Socially Inflexible and Demand Specific non-compliance were assessed by 24 items. Parents were first asked to rate if each situation is a problem on a *yes–no* scale, and if yes, the severity is rated on a 9-point scale from ‘*mild*’ to ‘*significant*’. The average severity rating per item was calculated.

Parent-reported child anxiety was measured using the Preschool Anxiety Scale Revised (PASR, Edwards et al. 2010). The PASR consisted of 28 statements tapping into specific fears, and generalized, social and separation anxiety which were rated on a 5-point scale ranging from ‘*not at all true*’ to ‘*very often true*’. A total score was calculated by summing the responses to all items, with higher scores indicating more anxiety.

### Teacher-Reported Child Emotional and Behavioral Problems

Teachers were asked to complete the Irritability and Hyperactivity subscales of the ABC (Aman et al. 1985).

### Parent-Reported Parenting Practices

Parent reports of their own parenting practices were measured using a shortened version of the Parenting Scale (PS, Arnold et al. 1993; Bodenmann et al. 2008). Lax (6 items) and Overreactive (5 items) parenting were rated on a 7-point scale anchored by opposing responses (e.g. when my child misbehaves, *I raise my voice and yell* [1]—*I speak to my child calmly* [7]. Higher scores on the two scales indicate more use of lax or overreactive parenting practices.

### Data Analysis

Data analysis was conducted in Stata 14 (StataCorp 2015). Descriptive statistics of the frequency and rate of the child (BTC; compliance) and parenting behaviors (facilitative; non-facilitative) were calculated. Because there were different tasks administered to minimally verbal children who formed a different population to verbal children, analyses were conducted by child verbal ability group. The distributions of the domain scores were examined to ensure that floor and ceiling effects were not present, as these might have restricted scope for identifying change. *T-*tests were used to examine differences by child verbal ability group. Item-level descriptive statistics are included in the supplementary materials.

To estimate inter-rater reliability (IRR), a random effects mixed model for unbalanced data using the *sem* command and maximum likelihood was used as we had multiple raters involved in rating different videos. In this model, the latent variable representing shared variance was estimated from the observed raters’ scores. Intraclass correlation coefficients (*ICC*s) examining the ratio of the variance in the latent variable over the variance in the latent variable + error variance were calculated (Koo and Li 2016) for the domain scores by child verbal ability group and 95% confidence intervals (*CI*) are presented. Item-level IRR was also examined and is presented in the supplementary materials. Although there is no agreed definition of adequate levels of inter-rater agreement and estimates vary depending on the method used, IRR estimates of greater than .70 or 70% agreement are generally acceptable (Aspland and Gardner 2003).

A multi-informant approach was used to explore the convergent validity of the OSCA–ABP child and parent domains. Convergent validity of the child BTC domain was explored using correlations to examine the associations between observed child behavior and parent- and teacher-reported child EBPs. Given the differences in context and sources of measurement error, as well as slight differences in the constructs that were being measured (i.e. BTC focuses on observable behavior that may challenge others, whereas EBPs encompasses a broader range of difficulties that may be present in different situations and measured over longer periods of time), we expected small to medium positive associations between observed child BTC and parent- and teacher-reported EBPs, in line with previous research (e.g. Wakschlag et al. 2008a, b). Observed child compliance was expected to be inversely associated with parent- and teacher-reported EBPs. No adjustment was made for multiple testing as we were interested in the patterns and directions of the relationships, and trends (*p* values below .10) and significant correlations are flagged.

Convergent validity of the parent domain scores was also examined by exploring the associations between observed parenting behaviors and parents’ reports of their own lax and overreactive parenting practices. Again, we expected correlations to be small given the differences in contexts, informants and constructs being assessed. We anticipated that more observed facilitative parenting behavior and a higher proportion of facilitative parenting would be associated with less self-reported lax and overreactive parenting, with the opposite pattern being seen for non-facilitative parenting. Again, no adjustment was made for multiple testing.

## Results

### Descriptive Statistics for the Sample and the OSCA–ABP Domains

**Fig. 1:**
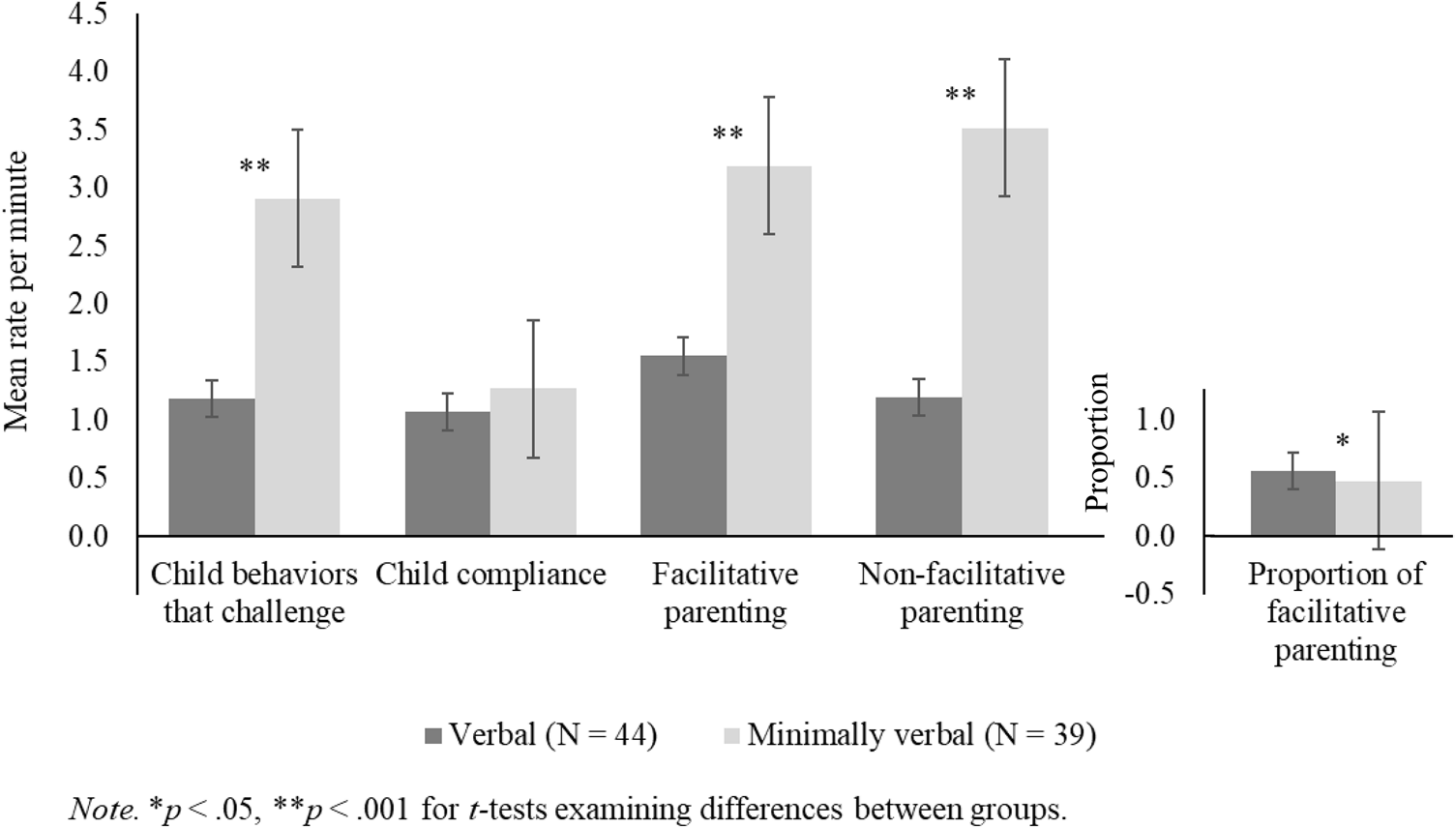
Graph showing mean rate per minute scores on the OSCA–ABP domains by child verbal ability group

Figure 1 shows the mean rates per minute on the OSCA–ABP domains by child verbal ability group. The descriptive statistics for the OSCA–ABP domain scores can be found in the supplementary materials. The mean duration of the observation was 20.5 min (*SD* = 1.7 min, range = 15.5–24.0 min). On average, both verbal and minimally verbal children displayed frequent BTC during the observation. Minimally verbal children displayed significantly more BTC than verbal children in the same time period (minimally verbal: *M* = 2.91 behaviors per minute, *SD* = 1.93 vs. verbal children: *M* = 1.18, *SD* = 1.22, *t*[81] = − 4.67, *p* < .001). All of the children in the current study exhibited some BTC during the observation. Eight verbal children (18.2% of the verbal sample), but no minimally verbal children, displayed fewer than five BTC. Child compliance was similar for verbal and minimally verbal children (see Fig. 1).

The frequency and rates of facilitative and non-facilitative parenting varied by child verbal ability group, with parents of minimally verbal children displaying twice as many parenting acts in the same time period (see Fig. 1 and the descriptive statistics in the supplementary materials). However, the proportion of facilitative parenting was similar for both groups of children and accounted for approximately half of all parenting behaviors. Correlations between the OSCA–ABP domains are in the Supplementary Materials.

### Inter-rater Reliability (IRR)

In order to establish IRR, 83 observations were coded from video-recordings by at least two of four raters (JPP, MF, SW, and EB), who wherever possible, were not involved in helping administer the observation. In addition, 23 of the videos were also coded by a third rater, resulting in a sample of 189 observations for IRR. The sample size and rating design was chosen to achieve 80% power. Coders were trained on observations obtained during initial pilot testing of the tasks and from post-intervention assessments during the feasibility stage. Training videos did not form part of the IRR sample. Meetings were held approximately every month to discuss any coding queries and achieve consensus, as well as to avoid coder drift.

Overall, IRR was excellent for the child BTC domain for both verbal and minimally verbal children (see Table 3 for *ICC*s and 95% *CI*). IRR was lower for child compliance, and IRR for facilitative parenting and non-facilitative parenting was good for both verbal and minimally verbal children. For a description of item-level IRR see the supplementary materials.

**Table 3:** ICCs for the OSCA–ABP domains by child verbal ability group

OSCA–ABP domains	Verbal (*N* = 44)	Minimally verbal (*N* = 39)
	*ICC* (95% *CI*)	*ICC* (95% *CI*)
Rate per minute scores		
Child behaviors that challenge rate	.92 (.88, .96)	.77 (.66, .89)
Child compliance rate	.45 (.25, .66)	.66 (.49, .82)
Facilitative parenting rate	.64 (.48, .80)	.75 (.63, .88)
Non-facilitative parenting rate	.67 (.52, .81)	.70 (.55, .85)
Proportion score		
Proportion of facilitative parenting	.68 (.54, .82)	.69 (.54, .84)
Frequency scores		
Child behaviors that challenge frequency	.92 (.87, 96)	.76 (.64, .88)
Child compliance frequency	.41 (.20, .63)	.64 (.47, .81)
Facilitative parenting frequency	.62 (.45, .79)	.77 (.65, .89)
Non-facilitative parenting frequency	.65 (.50, .81)	.71 (.58, .86)

### Convergent Validity

Table 4 below displays the correlations between the OSCA–ABP domains and parent- and teacher-reported child EBPs. Descriptive statistics for the parent and teacher questionnaires by child verbal ability are in the supplementary materials. More parent-reported child non-compliance on the HSQ-ASD was significantly associated with less observed child compliance during the OSCA–ABP in minimally verbal children (*r* =  − .35, *p* = .027). Small but significant correlations were found between teacher-reported child EBPs and observed child behavior among verbal children with more irritability (*r* = .36, *p* = .026) and hyperactivity (*r* = .34, *p* = .039) on the ABC being associated with more observed BTC. No significant relationships were found between teacher-reported child EBPs and observed child behavior for minimally verbal children. More observed non-facilitative parenting behaviors was associated with significantly more endorsement of lax parenting practices by parents of verbal children (*r* = .38, *p* = .010) and lower proportions of facilitative parenting was associated with more self-reported overreactive parenting (*r* =  − .35, *p* = .022). Among minimally verbal children, more observed facilitative parenting was associated with greater endorsement by parents of lax parenting practices (*r* = .34, *p* = .036).

**Table 4:** Convergent validity of OSCA–ABP domains by child verbal ability group

	Parent reported child emotional and behavioral problems^a^				Teacher reported child emotional and behavioral problems^b^		Parent reported parenting^a^	
	Irritability (ABC)	Hyperactivity (ABC)	Non-compliance (HSQ)	Anxiety (PASR)	Irritability (ABC)	Hyperactivity (ABC)	Lax parenting (PS)	Overreactive parenting (PS)
Verbal								
OSCA–ABP domains								
Child behaviors that challenge rate	.14	.28^†^	.12	− .19	.36*	.34*	–	–
Child compliance rate	− .20	− .05	− .06	.10	− .13	− .04	–	–
Facilitative parenting rate	–	–	–	–	–	–	.01	− .23
Non-facilitative parenting rate	–	–	–	–	–	–	.38*	.25
Proportion of facilitative parenting	–	–	–	–	–	–	− .26^†^	− .35*
Minimally verbal								
OSCA–ABP domains								
Child behaviors that challenge	.10	.04	.12	.16	.08	− .07	–	–
Child compliance rate	.03	− .22	− .35*	− .05	− .28	− .16	–	–
Facilitative parenting rate	–	–	–	–	–	–	.34*	.02
Non-facilitative parenting rate	–	–	–	–	–	–	.22	− .18
Proportion of facilitative parenting	–	–	–	–	–	–	.09	.21

Pearson’s *r* reported

*p < .05

^†^*p* < .10

^a^*N* = 43 for verbal children, parent-report measures missing for one child, *N* = 39 for minimally verbal

^b^*N* = 38 for verbal children, *N* = 38 for minimally verbal children

## Discussion

The current study aimed to establish the reliability and validity of a novel observational measure of child and parenting behavior in autism by examining the descriptive statistics of the measure, inter-rater reliability and convergent validity. Sufficient variability in child and parenting behavior was elicited by the measure. In contrast with previous research that has reported floor effects for child behavior on such measures used with autistic children who were screened in for moderate or greater EBPs (Bearss et al. 2015a; Handen et al. 2013), in general, floor effects were not found on the OSCA–ABP with high mean rates of BTC being observed by both verbal and minimally verbal children. All children in the current sample displayed at least one behavior that challenged during the observation and 90% displayed five or more behaviors. It appears that the selection and modification of tasks were effective in tapping into triggers that might underlie EBPs in autistic children. This supports the ecological validity of the measure, along with reports from parents indicating that behavioral displays seen during the assessment were typical of presentations in other environments and everyday situations. Despite the idiosyncratic triggers of EBPs seen in autism, it appears that by using a range of structured presses, child BTC is possible to elicit and observe in direct assessments. Use of this measure in the context of a trial would allow for blinded measurement of change in most children.

Of note were the differences in absolute frequencies of observed child BTC by child verbal ability group. In keeping with the literature (e.g. Einfeld et al. 2011; McClintock et al. 2003), minimally verbal children displayed significantly more BTC than verbal children. This is despite the absence of differences found in parent- and teacher-reported EBPs between groups. Although absolute levels of facilitative and non-facilitative parenting were also significantly higher among minimally verbal children, when the proportion of facilitative parenting behavior was examined, no group differences were found. It may be that the structure and set-up of the measure was more challenging for minimally verbal children resulting in more frequent BTC and parenting behavior. In naturalistic settings, parents of minimally verbal children may accommodate for and modify the environment in a way that was less possible whilst completing the tasks. This could include adapting expectations for their child and reducing demands, behaviors reportedly used by parents of children with intellectual disabilities (Green et al. 2014; Phillips et al. 2017). It is also possible that the unfamiliar environment may also be particularly anxiety-producing for minimally verbal children who have additional difficulties in communicating.

In addition to obtaining variability in responses on the measure, high inter-rater reliability for child BTC was demonstrated among both verbal and minimally verbal children. This adds to previous research (Handen et al. 2013; Wakschlag et al. 2008a, b) by demonstrating that child EBPs can be used observed reliably to quantify clinically salient disruptive and anxiety-related behaviors in young autistic children with and without intellectual disabilities. Despite anxiety-related behaviors being more difficult to measuring objectively, both verbal and minimally verbal children displayed avoidance and reassurance seeking during the observation. However, inter-rater reliability for items that aimed to measure anxiety was generally lower than items measuring externalising behavior. This is likely due to a reliance on assessing the child’s intention of their response to possible anxiety-provoking stimuli or situations. Furthermore, we removed one item that was originally intended to measure anxiety-related repetitive behaviors and vocalisations as it was difficult to distinguish when repetitive behaviors or vocalisations appeared to be an anxious response from an expression of autism (see supplementary materials). This is likely to have made high inter-rater reliability more difficult to achieve for this item. For parenting behaviors, intraclass correlation coefficients demonstrated adequate reliability. It was more difficult to obtain good inter-rater reliability for child compliance. This may be due to the requirement of first identifying a parental or researcher instruction for the child to comply with.

Initial convergent validity of the OSCA–ABP child domains was established. Among minimally verbal children, parent-reported non-compliance was associated with more observed child BTC, accounting for approximately 12% of the shared variance. These results are similar to other research with non-autistic children (e.g., Wakschlag et al. 2008a, b), where associations between observed disruptive behavior seen during a structured laboratory observation and parent-reports of disruptive behavior were weakly correlated. The convergent validity of the OSCA–ABP parent domains was also examined, and the general pattern of relationships between observed parenting and parent reports of their own parenting practices suggested that the OSCA–ABP constructs were valid, particularly among parents of verbal children. Literature examining the convergent validity of observations of parenting behavior has also reported associations in the low to moderate range (Hawes and Dadds 2006). However, the generalizability of such behavioral observations has been critiqued and it has been argued that the imposed structure of such assessments and presence of an observer may alter behavior (Rhule et al. 2009). It is also possible that observational measures are tapping into slightly different aspects of child and parenting behavior to parent reports. Parent reports may reflect more global child EBPs and parenting experiences, whereas during this measure, child and parent behavior is situation specific. Other possible factors that account for variation in agreement include different sources of measurement error reducing power to test relationships and broader rater effects, such as parental mental health problems (Najman et al. 2000).

In the current study, teacher reports of child EBPs were significantly associated with observed child BTC among verbal, but not minimally verbal children, accounting for between 12 and 13% of shared variance. Previous literature has reported small but significant associations between observed disruptive child behavior and teacher-reported EBPs in non-autistic samples (Wakschlag et al. 2008a, b). Furthermore, in line with literature in non-autistic populations, agreement between parent and teacher reports in autistic samples is modest (Stratis and Lecavalier 2017). It is possible that teachers of minimally verbal children, who were primarily employed in specialist education settings, have different thresholds for difficult child behavior which may influence their responses to questionnaire-based measures. Whereas, on the OSCA–ABP, raters reliably applied the same behavioral definitions across verbal and minimally verbal children. It is evident that verbal and minimally verbal children are two different populations and future research, possibly testing the measure in specialist education settings, to further understand these different patterns is warranted.

The measure’s sentivitiy to change was not explored in this manuscript and will be reported in a separate manuscript describing the findings from the trial. However, recent literature indicates that observational measures can pick up on changes in parenting behaviour following behavioral parenting interventions (e.g. Vetter 2018).

### Limitations

Several limitations should be noted. Test–retest reliability was not assessed and needs to be established in future research. In addition, although the tasks aimed to cover a range of potential triggers and were generally effective in eliciting child BTC, it is possible that idiosyncratic triggers that are clinically relevant may be missed in particular individuals. We also need to consider the applicability of the questionnaire measures used in the current study for autistic samples. Some of the measures used to establish convergent validity of the new observational measure were not developed for use in autism and/or intellectual disability, and some items may not be relevant or interpreted differently. There is also a possibility that autism-specific parenting behaviors often reported to manage child challenging behavior, anxiety and non-compliance (e.g. modifying the environment, limiting exposure to adverse sensory stimuli, reducing unpredictability by providing structure, routine and familiarity; O'Nions et al. 2018) were not identified using the parenting behavior coding scheme derived for the study and opportunities to demonstrate these parenting strategies may have been limited during the observation. Furthermore, the current coding scheme was developed to measure the frequency of child compliance, rather than the proportion of compliance with specific commands. Future versions of the measure may want to consider examining the reliability of coding compliance in relation to the amount and type of commands given by parents and researchers.

### Strengths

The study has a number of strengths. It describes the development and task selection of a new measure of observed child and parent behavior in autism with PPI input. Results indicate that the scheme for coding child and parenting behavior developed for use with this measure can be reliably applied to verbal and minimally verbal children and their parents. Reports from multiple informants were used to explore convergent validity giving support that the contrasts were valid and being adequately assessed. The use of the OSCA–ABP to identify difficulties of clinical relevance to families is promising.

### Conclusions

The promise of the OSCA–ABP for contributing to research and clinical practice in autism mental health beyond blinded measurement in trials is substantial. Co-occurring EBPs frequently exist in autism (e.g. Lai et al. 2019; Salazar et al. 2015; Simonoff et al. 2008) and current measures for examining these difficulties rely on parent or teacher reports. A particular challenge in research and clinical settings is obtaining accurate information about triggers and management strategies for EBPs. The OSCA–ABP could be used alongside other methods of assessment to obtain a more objective and comprehensive understanding of how child EBPs presents in autism and how parents interact with their children and respond to EBPs. As it is a standardized measure that is easy to administer, it has the potential to be useful in both research and clinical settings to assist with diagnosis of co-occurring difficulties in autism, clinical decision making regarding potential targets for intervention and as an outcome in trials.

## Electronic supplementary material

Below is the link to the electronic supplementary material.

Supplementary file1 (DOCX 88 kb)

## Footnotes

1 We use ‘autism’ to refer to Autism Spectrum Disorder (ASD; DSM-5) and ‘autistic children’ as this is the preferred term of some of the autism community (Kenny et al. 2016).
