# Supplementary material for: Development of the Observation Schedule for Children with Autism–Anxiety, Behaviour and Parenting (OSCA–ABP): A New Measure of Child and Parenting Behavior for Use with Young Autistic Children
Source: J Autism Dev Disord. 2020 Apr 30;51(1):1–14. doi: 10.1007/s10803-020-04506-3 (PMC7810641; doi:10.1007/s10803-020-04506-3)
Supplement: Supplementary file 1 — Supplementary file1 (DOCX 88 kb) [file 10803_2020_4506_MOESM1_ESM.docx]

**Supplementary Materials**

**Additional Information on the Coding Scheme Development**

In addition to frequency counts of the specific child and parenting behaviors, the presence and intensity of frustration and anxiety in each task is coded and amount of parental and child control is coded in tasks two, three, five, six and seven. Five global codes are used to rate the frequency and intensity of the child’s anxiety, disruptive behavior and aggression, hyperactivity and off-task behavior, and positive and negative affect on a 6-point Likert scale across the whole observation. The frequency and intensity of positive and negative affect in the parent and lax parenting across the whole observation is also rated on a 6-point Likert scale. As these codes are not included in the child and parent domain scores, details on IRR for these items are not included in this manuscript and further information can be provided by contacting the corresponding author.

As increased repetitive behavior and vocalizations are often reported as a behavioral manifestation of anxiety (Bearss et al., 2015; Ozsivadjian, Knott, & Magiati, 2012), the frequency of child repetitive behavior and vocalizations was coded but later omitted from the child BTC domain. This was due to concerns over differentiating anxiety-related repetitive behavior and vocalizations from core autistic characteristics in addition to obtaining highly negative skewed data for this item, primarily among minimally verbal children. Furthermore, parental attempts to scaffold and the frequency and success of scaffolding were coded. Due to difficulties in obtaining inter-rater reliability on these items and overlap with other codes such as clear commands and physical guidance, parental scaffolding is not included in the current facilitative parenting behavior domain score.

**Item-Level Inter-Rater Reliability (IRR)**

Item-level IRR for the child behavior items was also examined by child verbal ability group (see Table 3 below for means and standard deviations for each item). *ICC*s ranged from .42 (avoidance) to .98 (destructive behavior) for verbal children. The *ICC*s for five of the six items were above .67, indicating the specific child behaviors were in general coded reliably. Due to the low frequency of verbal aggression towards others and physical and verbal aggression towards oneself, the proportion of agreement between two raters was calculated for these items. For verbal children, the agreement ranged from 86.4-93.2%. Among minimally verbal children, item-level IRR was also high with *ICC*s for five of the six items being above .72 and ranged from .49 (avoidance) to .91 (destructive behavior). Agreement for verbal aggression towards others and physical and verbal aggression towards oneself ranged from 82.1% to 97.4%.

Item-level IRR for the specific parenting behaviors was also good, although slightly lower than child behavior. Amongst verbal children, *ICC*s ranged from .31 (positive comments) to .75 (negative comments) with seven of the nine items achieving *ICC* of above .56. For parents of minimally verbal children, *ICC*s were between .16 (positive comments) and .92 (non-descriptive praise), with *ICC*s for six of eight items being above .55. As parents of minimally verbal children tended to use less verbal strategies to communicate with their child, the lower *ICC* for comments may be related to more difficulty in identifying clear verbal statements. The proportion of agreement between two raters was calculated for the ninth item, descriptive praise, due to the low observed frequency of this behavior and agreement was 76.9%. Further details about the item-level descriptive statistics and reliability can be obtained from the corresponding author.

**Ecological Validity**

Ecological validity was explored by asking parents to comment briefly on how typical their child’s and their own behavior was during the observation. Parents described specific child behaviors that were seen during the observation as being typical of the child. These included the absence or presence of anxiety, physical aggression, self-injurious behavior, difficulties with attention and task shifting and compliance. Parents also described behaviors that they used in everyday situations that were observed during the assessment. For example, these included displaying facilitative parenting by encouraging, praising and adapting tasks and activities to their child’s needs, or reports of feeling frustrated or that the child was in control of the situation.

A key theme that was identified from parental feedback on the observation was the impact of the observed unfamiliar environment on both child and parenting behavior. Some parents described their child’s behavior (both disruptive and anxious behavior) as being worse than usual, e.g. “*she would be calmer at home and was anxious about her mother leaving the room*”*,* with this increase in BTC often being attributed to the new environment. Others described the opposite pattern, experiencing more BTC at home and suggesting the unfamiliar environment acted as a context to behave well. Parents also stated that the environment affected how they perceived their own parenting behavior and whether the impact was positive or negative. For instance, parents reported that their wariness of being filmed negatively affected their parenting self-efficacy.

Table 1. Coding definitions for items in the child and parent domain scores

| **OSCA–ABP domains** | | **Definition** |
| --- | --- | --- |
|  | **Item** |  |
| **Child behaviors that challenge** | |  |
|  | Destructive behavior | *Destructive Behavior* includes when the child *attempts* to damage or destroy an object or property. The deliberateness/intent of the disruptive behavior should be considered when coding the behavior.  Damage to an object or property should NOT be coded as *Destructive Behavior* if it results from clumsiness/poor motor skills. However, if a child has been advised to stop doing something because they would damage an object or property (e.g., engaging in a sensory interest with a toy), this IS to be coded as *Destructive Behavior*. Each act of *Destructive Behavior* should be coded as one event. For example, if the child slams the door twice in short succession, this should be coded as two events (rather than one episode of door slamming). |
|  | Physical aggression towards others | Behaviors that involve a physically aggressive act to the parent/carer or examiner. The child does NOT need to make contact with the individual for the act to be coded as a physically aggressive act (e.g. if child goes to slap the parent but they move away this should be coded as physical aggression). Each act of *Physical Aggression* to the parent/carer or examiner should be coded as one event. For example, if the child bites the parent/carer twice in short succession, this should be coded as two events (rather than one episode of biting). However, if the child bites the parent/carer once and does not let go immediately, this should be coded as one event. Self-Injurious behavior are NOT coded here as it is coded as *Physical Aggression Towards Self*. |
|  | Verbal aggression towards others | Vocalisations by the child that are verbally aggressive in nature directed towards to the parent/examiner. This would include cursing and using words to threaten another. Spiteful comments that aim to hurt another would be included here. Non-verbal behaviors that are aggressive in nature but not physical would be coded here (e.g., giving the finger). |
|  | Physical aggression towards self | Behaviors that involve an act or *attempt* of *Physical Aggression* to self. Each act of *Physical Aggression* to self should be coded as one event. For example, if the child bangs his head three times in a row, each bang is counted as an act and coded as three events (rather than one episode of three head bangs). |
|  | Verbal aggression towards self | Vocalisations by the child of a verbally aggressive nature consisting of a negative appraisal of themselves or their actions/performance on a task. Each vocalisation of should be coded as one event. |
|  | Frustrated vocalisations | Vocalisations by the child that are disruptive or negative in nature, irrespective of whether the content of the vocalisation. This includes vocalisations that express frustration. Vocalisations that are disruptive in nature but appear to be related to ASD symptomatology should **NOT** be coded here (e.g. squealing without apparent frustration or anxiety). Episodes of *Frustrated Vocalisations* are distinct if at least 1 second has lapsed between one episode of frustrated or disruptive vocalisations and the child displaying these behaviors again. |
|  | Non-compliance | When a child does NOT comply or *attempt* to comply with a reasonable command (both clear and unclear) from the parent/carer or examiner within 10 seconds of the command. Non-compliance could be active or passive. The child DOES need to have appeared to have heard the instruction for non-compliance, but there needs to be some opportunity for the child to be able to comply. |
|  | Avoidance | Behaviors that involve the child avoiding a perceived anxiety-provoking situation or physical and mental withdrawal from an anxiety-provoking situation. Episodes of *Avoidance* should be coded as one event. Episodes of *Avoidance* are distinct if at least 1 second has lapsed between one episode of avoidance and the child displaying these behaviors again. If a child attempts to leave the room during the separation, this should NOT be coded as *Avoidance*, and should be coded as *Reassurance Seeking*. |
|  | Reassurance seeking | Proximity seeking and reassurance seeking behaviors to parent/carer observed.  Episodes of *Reassurance Seeking* behavior are distinct if at least 1 second has lapsed between one episode of proximity and reassurance seeking behavior and the child displaying these behaviors again. For verbal *Reassurance Seeking* (e.g. saying where’s mum during separation), each instance should be coded as an event. |
|  |  |  |
| **Child compliance** | |  |
|  | Compliance | When a child complies or *attempts* to comply with a command (both clear and unclear) from the parent/carer or examiner within 10 seconds of the command. If the child complies but complains or whines whilst complying, this would still be coded as compliance. Presence of whining or complaining would also be coded under *Frustrated Vocalisations.* |
|  |  |  |
| **Facilitative parenting behavior** | |  |
|  | Positive comments | Verbal statements of love or affection of the child or enjoyment in spending time with child.  Each *Positive Comment* should be coded as one event, even if the comment is repeated. For example, ‘You’re such a lovely boy, SUCH a lovely boy’ should be coded as two events. |
|  | Clear command | A clearly stated *Command* that provides a specific instruction to the child in order to elicit a behavioral response (to do something or to stop doing something). The statement should be declarative in nature.  *Commands* can be coded as *Clear*, even if the verbal instruction by itself seems vague if it includes actions that make clear reference to an object or task. Obtaining the child’s eye contact is NOT necessary for a command to be considered *Clear*, but a reasonable attempt to capture the child’s attention should have been made, if not already engaged jointly with the parent in a task. *Commands* posed in question form should be coded as an *Unclear Command*. Comments that are informational in nature (about objects or events) that do not have an explicit intended response (e.g. ‘I’m going to draw the line this way’) should NOT be coded as commands. Comments that are informative in nature but do not explicitly describe an action (e.g., ‘Go on’, ‘Gently’) are NOT commands. Repeated instruction for the same command SHOULD be coded if they occur with 10 seconds of each other. If there is no opportunity to comply this should also be coded as *No Opportunity To Comply*. |
|  | Praise | Verbal reinforcement or verbal encouragement related to the child’s behavior or the child performance on a task. *Non-Specific Praise* is a vocalisation that expresses approval of the child, their behavior or performance on a task. Celebratory vocalisations (e.g. YEYY; woohoo) about child performance in a task should also be coded as *Non-Specific Praise*. To be coded as *Specific Praise*, the vocalisation must describe the behavior that to parent appreciates or trying to reinforce/encourage. *Specific Praise* is more desirable than *Non-Specific Praise*. Each expression of *Praise* should be coded as one event, even if the comment is repeated. For example, ‘Good boy, good boy’ should be coded as two events. Phrases which might sound like *Praise*, but may be coercive (e.g. ‘be a good boy’), should NOT be coded as praise but coded as a *Negative Comment*. |
|  | Physical guidance | Parent physically handling child in a NON forceful or aggressive manner (e.g. parent holds and guides child’s hand while writing). To be coded here, *Physical Guidance* should have the intent to physically help and/or support the child’s completion of tasks. Each *Physical Guidance* event displayed by the parent should be coded as a separate event if they can be differentiated from each other. If a parent gently holds the child in their seat and also guides the child’s hand while writing, this should be coded as two distinct *Physical Guidance* behaviors. If a parent takes their child’s hands and moves their legs to walk the line, this should be coded as one *Physical Guidance* behaviors. Acts of affection (e.g., hugging, kissing,) should NOT be coded as *Physical Guidance*. These should be considered when coding *Global* *Positive Affect*. |
|  |  |  |
| **Non-facilitative parenting behavior** | | |
|  | Negative comments | Verbal statements of disapproval or dislike of the child or the child’s performance in a task. This can include both task and non-task related behavior. Clear sarcastic comments would be coded here. Comments that are coercive but may sound like *Praise* ARE coded here. Each *Negative Comment* should be coded as one event, even if the comment is repeated. For example, ‘naughty’, ‘naughty’ should be coded as two events. |
|  | Unclear command | A *Command* given to a child that is stated in question form, vague or non-specific, that is intended to elicit a behavioral response. In order to be coded as *Commands* (either *Clear* or *Unclear*), there needs to be a specific intended behavioral response (see *Clear Commands* definition for further detail). Although content of the *Command* is important, the context in which the *Command* is given should be considered. As with *Clear Commands*, if the verbal instruction itself seems clear, but there has been no reasonable attempt to capture the child’s attention if not already engaged jointly in a task, or there is not clear reference, the *Command* should be considered *Unclear*. Questions that request an answer but do not intend to elicit a behavioral response from the child should NOT be coded as *Commands* (e.g. ‘Do you want to use that dial?’). |
|  | No opportunity to comply | When a child is not given the opportunity to comply with a reasonable command (both clear and unclear) from the parent/carer or examiner within 10 seconds of the command. *No Opportunity To Comply* may be because the parent repeated the command within 10 seconds or replaced the command with a second or overriding one, the parent acted without giving the child an opportunity to comply or the parent used physical action to make the child comply. Unreasonable commands (expectations outside of developmental capabilities, requiring major parental intervention to be completed) should be coded as *No Opportunity To Comply.* If the child turns away from parent/carer in response to a command and the parent repeats the command, this should be coded as *Non-Compliance*. However, if the parent repeats the command within the 10 seconds window and before any action was taken by the child, this would be coded as *No Opportunity To Comply*. |
|  | Physical handling | Parent physically handling child in a rough or controlling way (e.g. parent grabs and pulls child unnecessarily). To be coded here, *Physical Handling* needs to be forceful or aggressive in nature, even if it facilitates the child to complete a task. This can include physical restraint if the coder thinks that it was overly aggressive in its nature. It also includes interference with a child’s ongoing activity if there is no concern for the child’s safety, safety of another person or destruction of an object (e.g. snatching, blocking access). If rough physical handling evolves into physical facilitation of completing a task, this should be coded as both an instance of *Physical Handling* and *Physical Guidance*. Each *Physical Handling* displayed by the parents should be coded as a separate event if they can be differentiated from each other. If a parent grabs their child and forcefully put them in the chair, this should be coded as two distinct *Physical Handling* behaviors. If a parent grabs their child, then releases them and immediately grabs them again, this should be coded two distinct *Physical Handling* behaviors. |
|  |  |  |
| **Removed items** | |  |
|  | Child repetitive behavior and vocalisations (originally in child behaviors that challenge) | Child behaviors or vocalisations that are unusually repetitive or excessive. To be coded here, the *Repetitive Behavior/Vocalisation* needs to have an anxious component to it. Self-injurious behaviors that have a repetitive nature should **NOT** be coded here and should be coded as *Physical Aggression Towards Self*. Anxious repetitive behaviors (e.g. rocking) that evolves into unintentional injury should NOT be coded as *Physical Aggression Towards Self* - how the behavior starts should define the behavior. However, if repetitive behavior evolves into intentional harm to self, this SHOULD be coded as both a *Repetitive Behavior* and *Physical Aggression Towards Self*. Each *Repetitive Behaviors/Vocalisations* should be coded as one event. |
|  | Parental scaffolding | Parental behaviors that provide help and support to help the child’s completion of tasks, verbally or non-verbally. This includes parents supporting child’s completion by adapting the task to suit the child’s interests/needs provided that the aim of the press is not altered. An attempt at facilitation does NOT need to result in child completion of task to be coded. The parent needs to give the child the opportunity to do the behavior/task independently (at least 10 seconds), rather than take control of the task to ensure completion. Reassurance from the parent can be coded here if it appears to encourage the child to continue with a task. Efforts to facilitate predictability should be coded here (e.g. explaining to child what will happen during a task or transition: ‘Now we are playing with the Etch-a-Sketch, next we’ll play with the Bop-It’). Each *Scaffolding* attempt displayed by the parents should be coded as a separate event if they can be differentiated from each other. If a parent takes shoes off and walks the line to model the whole task for the child, this would be coded as one *Scaffolding* event. If a parent aims for the child to take off their shoes and models it by taking shoes off, and subsequently aims for the child to walk the line and explains how to do it properly, this should be coded two distinct *Scaffolding* behaviors. Physical support to complete a task (e.g. taking hand and guiding it through writing) is NOT coded here, but in *Physical Guidance*. |
| © King’s College London. Version 2.0, dated 20/11/2017. | | |

Table 2. Descriptive statistics for the OSCA–ABP domains by child verbal ability group

| **OSCA–ABP domains** | **Verbal (*N* = 44)** | | |  | **Minimally verbal (*N* = 39)** | | |
| --- | --- | --- | --- | --- | --- | --- | --- |
|  | ***M*** | ***SD*** | **Observed range** |  | ***M*** | ***SD*** | **Observed range** |
| **Rate per minute scores** |  |  |  |  |  |  |  |
| Child behaviors that challenge rate** | 1.18 | 1.22 | 0.05-5.23 |  | 2.91 | 1.93 | 0.35-8.74 |
| Child compliance rate | 1.07 | 0.45 | 0.39-2.34 |  | 1.27 | 0.72 | 0.06-3.01 |
| Facilitative parenting rate** | 1.55 | 0.79 | 0.47-4.06 |  | 3.19 | 1.29 | 1.17-6.29 |
| Non-facilitative parenting rate** | 1.19 | 0.69 | 0.23-2.94 |  | 3.52 | 1.51 | 1.20-6.16 |
|  |  |  |  |  |  |  |  |
| **Proportion score** |  |  |  |  |  |  |  |
| Proportion of facilitative parenting* | 0.57 | 0.14 | 0.22-0.88 |  | 0.48 | 0.12 | 0.23-0.79 |
|  |  |  |  |  |  |  |  |
| **Frequency scores** |  |  |  |  |  |  |  |
| Child behaviors that challenge frequency** | 24.82 | 25.91 | 1-107 |  | 57.59 | 37.58 | 7-171 |
| Child compliance frequency | 22.14 | 8.70 | 7-46 |  | 25.00 | 13.55 | 1-52 |
| Facilitative parenting frequency** | 32.32 | 16.02 | 10-79 |  | 63.56 | 26.34 | 24-123 |
| Non-facilitative parenting frequency** | 24.93 | 14.37 | 5-62 |  | 70.14 | 31.20 | 24-135 |
| *Note.* **p* < .05, ***p* < .001 for *t* tests examining differences between groups. | | | | | | | |

Table 3. Descriptive statistics for the OSCA–ABP items by child verbal ability group

| **OSCA–ABP domains** | | **Verbal (*N* = 44)** | | |  | **Minimally verbal (*N* = 39)** | | |
| --- | --- | --- | --- | --- | --- | --- | --- | --- |
|  |  | ***M*** | ***SD*** | **Observed range** |  | ***M*** | ***SD*** | **Observed range** |
| **Child behaviors that challenge** | |  |  |  |  |  |  |  |
|  | Destructive behavior | 3.09 | 9.96 | 0-65 |  | 4.54 | 11.68 | 0-69 |
|  | Physical aggression towards others | 1.00 | 2.24 | 0-12 |  | 3.32 | 4.60 | 0-23 |
|  | Verbal aggression towards others | 0.07 | 0.33 | 0-2 |  | 0.00 | 0.00 | 0 |
|  | Physical aggression towards self | 0.25 | 0.81 | 0-4 |  | 1.36 | 5.98 | 0-37 |
|  | Verbal aggression towards self | 0.30 | 0.95 | 0-4 |  | 0.00 | 0.00 | 0 |
|  | Frustrated vocalisations | 8.64 | 11.04 | 0-46 |  | 18.95 | 22.13 | 0-118 |
|  | Non-compliance | 5.75 | 7.72 | 0-36 |  | 13.21 | 8.09 | 0-31 |
|  | Avoidance | 3.77 | 4.73 | 0-19 |  | 10.59 | 7.02 | 0-34 |
|  | Reassurance seeking | 1.95 | 2.37 | 0-8 |  | 5.63 | 6.74 | 0-28 |
|  |  |  |  |  |  |  |  |  |
| **Facilitative parenting behavior** | |  |  |  |  |  |  |  |
|  | Positive comments | 2.77 | 2.09 | 0-9 |  | 2.12 | 2.67 | 0-13 |
|  | Clear command | 13.02 | 7.68 | 2-30 |  | 29.81 | 17.49 | 4.57-73 |
|  | Non-specific praise | 13.02 | 9.98 | 1-51 |  | 20.76 | 15.71 | 0-76 |
|  | Specific praise | 0.89 | 1.50 | 0-6 |  | 0.72 | 1.57 | 0-7 |
|  | Physical guidance | 2.61 | 3.51 | 0-19 |  | 10.14 | 6.68 | 0-29 |
|  |  |  |  |  |  |  |  |  |
| **Non-facilitative parenting behavior** | |  |  |  |  |  |  |  |
|  | Negative comments | 0.91 | 1.65 | 0-9 |  | 2.70 | 4.41 | 0-22 |
|  | Unclear command | 17.39 | 9.75 | 3-44 |  | 35.38 | 13.17 | 17-65 |
|  | No opportunity to comply | 5.93 | 5.58 | 0-21 |  | 26.29 | 16.20 | 4.57-60 |
|  | Physical handling | 0.70 | 1.27 | 0-4 |  | 5.78 | 5.78 | 0-24 |

Table 4. Table showing correlations between OSCA–ABP domains by child verbal ability group

| **OSCA–ABP domains** | **Verbal (*N* = 44)** | | | |  | **Minimally verbal (*N* = 39)** | | | |
| --- | --- | --- | --- | --- | --- | --- | --- | --- | --- |
|  | 1. | 2. | 3. | 4. |  | 1. | 2. | 3. | 4. |
| 1. Child behaviors that challenge frequency | - |  |  |  |  | - |  |  |  |
| 1. Child compliance frequency | -.11 | - |  |  |  | -.14 | - |  |  |
| 1. Facilitative parenting frequency | .29† | .54** | - |  |  | .05 | .67** | - |  |
| 1. Non-facilitative parenting frequency | .50** | .42* | .42* | - |  | .01 | .41* | .39* | - |
| 1. Proportion of facilitative parenting | -.19 | -.13 | .33* | -.65** |  | -.05 | .24 | .54** | -.53** |
| *Note.* Pearson’s *r* reported. †*p* < .10, **p* < .05, ***p* < .001. | | | | | | | | | |

Table 5. Participant characteristics by child verbal ability group

| **Sample characteristics** | | **Verbal (*N* = 44)** | | |  | **Minimally verbal (*N* = 39)** | | |  |  |
| --- | --- | --- | --- | --- | --- | --- | --- | --- | --- | --- |
|  |  | *M*  or *n* | *SD*  or % | Observed  range |  | *M*  or *n* | *SD*  or % | Observed  Range |  | Possible range |
| Autism severity | |  |  |  |  |  |  |  |  |  |
|  | ADOS–2 CSS total | 7.45 | 2.37 | 1-10 |  | 7.62 | 1.21 | 5-10 |  | 1-10 |
|  | ADOS–2 classification (ASD or autism)^a^ (*n* and %) | 40 | 90.9% |  |  | 39 | 100.0% |  |  |  |
|  | Parent reported SCQ-L total^c^ | 22.56 | 7.36 | 9-37 |  | 24.41 | 5.80 | 12-34 |  | 0-40 |
|  | SCQ-L classification (ASD or autism)^a,c^ (*n* and %) | 36 | 83.7% |  |  | 37 | 94.9% |  |  |  |
|  |  |  |  |  |  |  |  |  |  |  |
| Level of speech (ADOS-2 module administered) | |  |  |  |  |  |  |  |  |  |
|  | Module 1, pre-verbal or single words (*n* and %) | 0 | 0.0% |  |  | 38 | 97.4% |  |  |  |
|  | Module 2, phrase speech (*n* and %) | 6 | 13.6% |  |  | 1 | 2.6% |  |  |  |
|  | Module 3, fluent speech (*n* and %) | 38 | 86.4% |  |  | 0 | 0.0% |  |  |  |
|  |  |  |  |  |  |  |  |  |  |  |
| Adaptive behavior^c^ | |  |  |  |  |  |  |  |  |  |
|  | ABAS–3 General Adaptive Composite (GAC) standard score** | 71.14 | 10.48 | 51-100 |  | 53.00 | 7.06 | 45-73 |  | 10-166^e^ |
|  | ABAS–3 GAC classification (≥70)^b^ (*n* and %)** | 22 | 51.2% |  |  | 2 | 5.1% |  |  |  |
|  |  |  |  |  |  |  |  |  |  |  |
| Parent reported child EBPs | |  |  |  |  |  |  |  |  |  |
|  | Parent reported child irritability (ABC irritability total)^c^ | 16.35 | 10.62 | 0-37 |  | 16.38 | 10.08 | 1-44 |  | 0-45 |
|  | Parent reported child hyperactivity (ABC hyperactivity total)^c^ | 23.58 | 14.79 | 1-48 |  | 24.64 | 11.71 | 8-48 |  | 0-48 |
|  | Parent reported child non-compliance (HSQ-ASD mean item severity)^c^ | 3.26 | 1.93 | 0.17-7.75 |  | 3.26 | 2.03 | 0.67-8.83 |  | 0-9 |
|  | Parent reported child anxiety (PASR total)^c^* | 47.02 | 24.55 | 0-91 |  | 33.21 | 18.18 | 6-95 |  | 0-112 |
|  |  |  |  |  |  |  |  |  |  |  |
| Teacher reported child EBPs | |  |  |  |  |  |  |  |  |  |
|  | Teacher reported child irritability (ABC irritability total)^d^ | 8.95 | 10.74 | 0-39 |  | 9.37 | 9.51 | 0-44 |  | 0-45 |
|  | Teacher reported child hyperactivity (ABC hyperactivity total)^d^ | 14.95 | 11.39 | 0-38 |  | 18.92 | 13.39 | 1-47 |  | 0-48 |
|  |  |  |  |  |  |  |  |  |  |  |
| Parent reported parenting | |  |  |  |  |  |  |  |  |  |
|  | Parent reported lax parenting (PS Lax total)^c^ | 2.84 | 1.06 | 1.33-6.17 |  | 3.10 | 0.91 | 1.5-4.5 |  | 1-7 |
|  | Parent reported overreactive parenting (PS Overreactive total)^c^ | 2.86 | 1.01 | 1.2-5.2 |  | 2.51 | 0.93 | 1-4.6 |  | 1-7 |
| *Note.* ABAS–3=Adaptive Behavior Assessment System – 3^rd^ edition; ABC=Aberrant Behavior Checklist; ADOS–2=Autism Diagnostic Observation Schedule – 2^nd^ edition; HSQ-ASD=Home Situations Questionnaire-Autism Spectrum Disorders; PASR= Preschool Anxiety Scale Revised; PS= Parenting Scale; SCQ=Social Communication Questionnaire.  ^a^Remainder non-spectrum.  ^b^Remainder <70.  ^c^*N* = 82, parent-report measures missing for one child (43 verbal, 39 minimally verbal).  ^d^*N* = 76 (38 verbal, 38 minimally verbal).  ^e^For age group.  **p* < .05, ***p* < .001 for *t* tests / χ^2^ tests examining differences between groups. | | | | | | | | | | |
